# Condensin I and condensin II proteins form a LINE-1 dependent super condensin complex and cooperate to repress LINE-1

**DOI:** 10.1093/nar/gkac802

**Published:** 2022-09-28

**Authors:** Jacqueline R Ward, Afshin Khan, Sabrina Torres, Bert Crawford, Sarah Nock, Trenton Frisbie, John V Moran, Michelle S Longworth

**Affiliations:** Department of Inflammation and Immunity, Lerner Research Institute, Cleveland Clinic, Cleveland, OH 44195, USA; Department of Inflammation and Immunity, Lerner Research Institute, Cleveland Clinic, Cleveland, OH 44195, USA; Department of Inflammation and Immunity, Lerner Research Institute, Cleveland Clinic, Cleveland, OH 44195, USA; Department of Inflammation and Immunity, Lerner Research Institute, Cleveland Clinic, Cleveland, OH 44195, USA; Department of Genetics and Genome Sciences, Case Western Reserve University, Cleveland, OH 44195, USA; Department of Human Genetics, University of Michigan School of Medicine, Ann Arbor, MI 48109, USA; Department of Human Genetics, University of Michigan School of Medicine, Ann Arbor, MI 48109, USA; Internal Medicine, University of Michigan School of Medicine, Ann Arbor, MI 48109, USA; Department of Inflammation and Immunity, Lerner Research Institute, Cleveland Clinic, Cleveland, OH 44195, USA; Cleveland Clinic Lerner College of Medicine, Case Western Reserve University School of Medicine, Cleveland, OH 44195, USA

## Abstract

Condensin I and condensin II are multi-subunit complexes that are known for their individual roles in genome organization and preventing genomic instability. However, interactions between condensin I and condensin II subunits and cooperative roles for condensin I and condensin II, outside of their genome organizing functions, have not been reported. We previously discovered that condensin II cooperates with Gamma Interferon Activated Inhibitor of Translation (GAIT) proteins to associate with Long INterspersed Element-1 (LINE-1 or L1) RNA and repress L1 protein expression and the retrotransposition of engineered L1 retrotransposition in cultured human cells. Here, we report that the L1 3′UTR is required for condensin II and GAIT association with L1 RNA, and deletion of the L1 RNA 3′UTR results in increased L1 protein expression and retrotransposition. Interestingly, like condensin II, we report that condensin I also binds GAIT proteins, associates with the L1 RNA 3′UTR, and represses L1 retrotransposition. We provide evidence that the condensin I protein, NCAPD2, is required for condensin II and GAIT protein association with L1 RNA. Furthermore, condensin I and condensin II subunits interact to form a L1-dependent super condensin complex (SCC) which is located primarily within the cytoplasm of both transformed and primary epithelial cells. These data suggest that increases in L1 expression in epithelial cells promote cytoplasmic condensin protein associations that facilitate a feedback loop in which condensins may cooperate to mediate L1 repression.

## INTRODUCTION

Condensin I and condensin II are multi-subunit protein complexes that play fundamental roles in chromosome organization and mitotic chromosome segregation (1–7) and also regulate innate immune responses (8,9). Mammalian cells possess two condensin complexes, condensin I and condensin II (7,10), that differ in their protein components and cellular localization during the cell cycle. Condensin I and condensin II contain two Structural Maintenance of Chromosomes (SMC) subunits, SMC2 and SMC4, which form the enzymatic (ATPase) and structural core of the complex. Unique to each condensin complex are the three non-SMC subunits or Chromatin Associated Proteins (CAPs). Condensin I contains NCAPD2, NCAPG, and the kleisin family protein, NCAPH. Condensin II contains NCAPD3, NCAPG2 and the kleisin family protein, NCAPH2. The CAP proteins stabilize the condensin holocomplex, promote ATPase activity, and associate with histones (11–17). Under normal cellular conditions, condensin II is found in the nucleus and cytoplasm throughout the cell cycle. Condensin I is predominantly cytoplasmic and contacts DNA after nuclear envelope breakdown during mitosis (7,18,19). Recent studies suggest condensin proteins also regulate chromosome organization and gene expression in interphase nuclei of vertebrate cells (3,20–22) and may play important roles in DNA repair (reviewed in (23)). The loss of condensin protein expression has been linked to cancers and developmental disorders (24–30).

Although the nuclear roles of condensin proteins are well studied, little is known about their cytoplasmic functions. We recently demonstrated that the condensin II complex represses the expression of endogenous Long INterspersed Element-1 (LINE-1 or L1) proteins and the retrotransposition of engineered human L1s in human epithelial cells (31). L1-derived-sequences comprise ∼17% of the human genome (32). However, the average human genome only contains ∼100 autonomously active full-length L1s that can mobilize to new genomic locations (33,34) via an RNA intermediate by a copy-and-paste mechanism known as retrotransposition (32,35,36). L1 retrotransposition requires the transcription of a full-length L1 mRNA, translation of its encoded proteins (ORF1p and ORF2p), and subsequent insertion of L1 mRNA into the genome via target-primed reverse transcription (TPRT) (37–39). L1 retrotransposition can generate genomic instability (38,40–43) and, on rare occasions, can cause diseases, including hemophilia A, Duchenne muscular dystrophy, and cancers [e.g. (44–53)]. Condensin II associates with the Gamma Interferon Activated Inhibitor of Translation (GAIT) complex and inhibits the binding of the translation initiation complex to L1 mRNA, thereby reducing the accumulation of L1 proteins essential for retrotransposition (31). GAIT originally was discovered as a translational suppressor of inflammation-related mRNAs in monocytes in response to IFNγ (54–56). However, the identities of the proteins and signaling pathways that regulate condensin II/GAIT association with L1 mRNA require elucidation.

Here, we report that the L1 RNA 3′UTR is necessary for condensin II and GAIT association with L1 RNA. We further show that Condensin I also associates with the L1 RNA 3′UTR and may mediate condensin II and GAIT association with L1 RNA. Finally, we demonstrate that, in response to L1 expression, subunits of the condensin I and condensin II complexes interact to form a primarily cytoplasmic, L1 3′UTR independent, Super Condensin Complex (SCC) in response to L1 expression. Together, these data uncover potential cooperative roles for cytoplasmic condensin complexes in the inhibition of L1 expression and retrotransposition.

## MATERIALS AND METHODS

### Cell lines

All mammalian cell culture lines were obtained from the American Type Culture Collection (ATCC). HT-29 cells (HTB-38) were cultured in RPMI 1640 media supplemented with 10% fetal bovine serum (FBS; Life Technologies) and 1% penicillin/streptomycin (Gibco). The hTERT RPE-1 cells (CRL-4000; written as RPE-1 for the remainder of the manuscript) were cultured in DMEM media supplemented with 10% FBS (Life Technologies) and 1% penicillin/streptomycin (Gibco). Cells were grown in a 37°C incubator with 5% CO_2_ levels.

### Preparation of cell lines expressing inducible small hairpin RNAs

Lentiviral transduction was performed in HT-29 cells using custom viral particles produced with the pLKO-puro-IPTG-3xLacO vector (Sigma-Aldrich). As described previously (57), in the absence of the lactose analog isopropyl-β-D-thio-galactoside (IPTG) the lactose repressor protein, LacI, binds to the lactose operator sequence (LacO) to prevent short hairpin RNA (shRNA) expression. In the presence of IPTG, a conformational change in the allosteric LacI repressor releases it from the LacO modified human U6 snRNA promoter allowing shRNA expression.

Briefly, HT-29 cells were plated in a 12-well tissue culture plate using 1 ml of media/well and incubated overnight in a 37°C incubator with 5% CO_2_. The following day, growth medium was prepared containing 8μg/ml of polybrene and was added to each well. HT-29 cell lines expressing Non-target (NT) and NCAPD3 shRNAs were previously described (57). To create the additional HT-29 shRNA expressing cell lines, NCAPD2 or NCAPG shRNA lentiviral particles (50μl, 1000 viral particles/μl) were added to the cells, gently mixed, and incubated at 37°C for 8 hours. After incubation, the polybrene containing media was changed to normal growth media. Three days post-infection, cells were selected with puromycin (12μg/ml) and quantitative reverse transcriptase PCR (qRT-PCR) and immunoblot analysis were used to assay the resultant stable clonal cells lines for decreased RNA and protein expression.

### Plasmid constructs

#### pCEP4

A mammalian expression vector (Life Technologies) used to construct some L1 expression plasmids (indicated below). The plasmid is augmented with a cytomegalovirus (CMV) immediate early promoter and an SV40 polyadenylation signal. The plasmid backbone also contains a hygromycin resistance selectable gene (*HYG*), the Epstein Barr Virus Nuclear Antigen-1 gene (*EBNA-1*), and *cis*-acting sequences (oriP) required for plasmid replication in human cells.

#### pJM101/L1.3

A pCEP4-based plasmid that contains an active human L1 (L1.3) equipped with a *mneoI* retrotransposition indicator cassette in its 3′ UTR (33,58).

#### pJM101/L1.3 3′UTRΔ

A pCEP4-based plasmid that contains a version of pJM101/L1.3 harboring the sequence [5′-ACAATGAGTTTAAACGTATACATATGTAACTAA-3′] in the place of the full-length 3′UTR (see Supplementary Figure S1).

### Antibodies

The following primary antibodies were utilized throughout this study for immunoblot analyses, immunoprecipitation experiments, or Proximity Ligation Assays: NCAPD3 (Bethyl Laboratories, catalog #A300-604A), NCAPD2 (Bethyl Laboratories, catalog #A300-601A and Santa Cruz Biotechnology, catalog #sc-166878), NCAPG2 (Abcam, catalog #ab70350), NCAPG (Bethyl Laboratories, catalog #A300-602A), EPRS (Bethyl Laboratories, catalog #A303-957A), eIF4G (Santa Cruz Biotechnology, catalog #sc-11373), Actin (Millipore, catalog #MAB1501), Normal Rabbit IgG (Millipore, catalog #12–370), SMC4 (Bethyl Laboratories, catalog #A300-063A), SMC2 (Bethyl Laboratories, catalog #A300-058A), NCAPH2 (Bethyl Laboratories, catalog #A302-276A), β-tubulin (Cell Signaling, catalog #2146). Purified polyclonal α-ORF1p was generated by OpenBiosystems and characterized in the laboratory of John V. Moran (University of Michigan School of Medicine) (59,60).

### Cell-culture based retrotransposition assay

Retrotransposition assays were performed as described previously with minor modifications (35,61–63). Briefly, for G418-resistance–based retrotransposition assays in HT-29 cells, ∼8 × 10^4^ per well were seeded into two sets of six-well plates tissue culture plates (Corning). For assays involving shRNA mediated knockdown of condensin proteins, clonally expanded cell lines described above were treated with IPTG for 48 hours to induce shRNA expression. Subsequently (and in experiments that did not involve shRNA induction), cells were transfected with 3 μg of the indicated L1 expression plasmid using 8 μl of the Lipofectamine 2000 transfection reagent (Invitrogen) per well. Seventy-two hours after transfection, cells were collected from one set of plates and the pellets were frozen at -80°C for future analysis. Cells from the other set of plates were treated with 0.05% trypsin/0.53 mM EDTA and resuspended in complete RPMI medium supplemented with G418 (600μg/ml) (Life Technologies). Cells from each well were plated onto three 10-cm tissue culture dishes, generating triplicate cultures. After 10 days of G418 selection, the remaining cells were treated with 5 mL of 10% formaldehyde in PBS at room temperature for 5 min to fix them to tissue culture plates, stained with 0.05% crystal violet in PBS for 30 min, washed twice with PBS, and imaged with a ChemiDoc XRS+ (Bio-Rad). The images were analyzed using ImagePro Plus 7.0 software. For one 10-cm dish, a circular area of interest (AOI), which delimits the population of pixels in the image was made. The same AOI was used as a template for each subsequent image analyzed. The number of drug-resistant foci were determined within the AOI and recorded for statistical analysis.

### Immunoblotting and Immunoprecipitation Analyses

Immunoprecipitation and immunoblotting to detect proteins of interest were performed as described previously (31). Bands were quantified from films, using a BioRad ChemiDoc™ XRS + Molecular Imager and Image Lab™ software with ‘Protein Gel’ settings. Identically sized windows were used to calculate Absolute volumes following the protocol provided by the manufacturer. Intensity values of all bands were first normalized to respective loading controls prior to comparisons between lanes.

### RNA analysis by quantitative reverse transcriptase PCR (qRT-PCR)

Cells were lysed and total RNAs were extracted using the Trizol reagent (Life Technologies). The RNAs were then treated with RNase-free DNase in buffer RDD (Qiagen) prior to further purification using a RNeasy Mini Kit (Qiagen). An aliquot of cDNA was generated from 1–2 μg of total RNA using the TaqMan Reverse Transcription Reagents (Applied Biosystems) and an oligo-dT(16) primer (Invitrogen). Quantitative RT-PCR was performed using the Roche Lightcycler 480 to amplify 15 μl reactions containing .5 μl of cDNA, .5 μl of a 10 μM primer mix and 7.5 μl of Fast Start SYBR Green Master Mix (Roche). Each reaction was performed in triplicate. Crossing point (Cp/ Ct) values were determined using the Roche LightCycler 480 Absolute Quantification Second Derivative Analysis software. Relative quantitation of transcript levels was then performed using the delta delta Ct method (2^−ΔΔCt^) where the Ct values of a reference gene (*actin*) in each sample are subtracted from the Ct values of the gene of interest to create a ΔCt value for each sample. The ΔCt is compared to a control sample to generate a ΔΔCt value for each sample. Following calculation of 2^−ΔΔCt^ for each sample, triplicates were averaged. Three, independent biological replicates were performed in all experiments, and the results of the three assays were averaged together, and standard deviations were calculated. L1 primer sequences were previously published (31). The sequences of oligos used in the qRT-PCR studies are listed below:

*L1 5′UTR* FW: 5′-ACGGAATCTCGCTGATTGCTA-3′

*L1 5′UTR* RV: 5′-AAGCAAGCCTGGGCAATG-3′

*L1 ORF1* FW: 5′-TCAAAGGAAAGCCCATCAGACTA-3′

*L1 ORF1* RV: 5′-TGGCCCCCACTCTCTTCT-3′

*Actin* FW: 5′-CCAACCGCGAGAAGATGACC-3′

*Actin* RV: 5′-GGAGTCCATCACGATGCCAG-3′

### RNA Immunoprecipitation (RIP)/RT-PCR assay for L1 RNA binding

The associations between NCAPD3, NCAPD2, NCAPG, EPRS, eIF4G and L1 RNA in HT-29 cells were evaluated using a ribonucleoprotein immunoprecipitation assay involving formaldehyde crosslinking, as described previously (31,64,65). Briefly, 6 × 10^6^ cells were seeded onto 150 mm tissue culture dishes and transfected with 40 μg pJM101/L1.3 or pJM101/L1.3 3′UTRΔ construct, using 100 μl Lipofectamine and following the manufacturer's protocol. Transfected cells were incubated with the transfection mix in complete media without penicillin/streptomycin, for 6 h, followed by a change to complete media with penicillin/streptomycin. Twenty-four hours post-transfection, Protein A Dynabeads (Invitrogen) were washed twice with cold PBS and then incubated with 6 μg of NCAPD2, NCAPD3, NCAPG, EPRS or eIF4G antibody, overnight, at 4°C, on a rocker, followed by two additional washes with cold PBS. Forty-eight hours post-transfection, cells were harvested and crosslinked in 1% formaldehyde in PBS for 10 minutes at room temperature, with rotation. 0.125 M glycine, pH 2.8 was added to quench the reaction; cells were incubated in this solution for 10 min at room temperature, with rotation. Cells were washed in PBS and resuspended in high salt lysis buffer (300 mM NaCl, 50 mM Tris, pH 7.5, 1 mM EDTA, 0.1% Triton-X 100, 10% glycerol, 1 mM DTT, Complete mini-protease inhibitor cocktail without EDTA (Sigma); 0.5 ml buffer per 6 × 10^6^ cells was used in each experiment). Lysates were then dounce homogenized 10 times and centrifuged at 14 000g for 15 min at 4°C. Each set of transfections was pooled together and re-aliquoted into three tubes per experimental condition. Lysates (0.5 ml) were pre-cleared with 50 μl of a Protein A Dynabead slurry containing 50% cold PBS and 50% Protein A Dynabeads that had been washed 2× with cold PBS. Preclearing was performed on a rocker at 4°C for 1 h. Lysates were then incubated with the Dynabead-antibody mixtures overnight, at 4°C, on a rocker. Beads were then washed once with high salt lysis buffer for 10 minutes, at 4°C, on a rocker, and once with cold PBS for 10 min, at 4°C, on a rocker. One hundred μl of beads were removed and boiled with 25 μl Laemmli buffer for 10 min; these protein samples were used to perform immunoblotting. Eighty μl of 50 mM glycine, pH 2.8, was then added to the remaining beads, and this solution was heated at 70°C for 45 min to reverse crosslinks. Trizol LS (Ambion, Life Technologies) was added to the supernatants, and RNA was isolated according to the manufacturer's protocol. RNA was purified on an RNA-easy column, including an on-column DNAse digestion (Qiagen); cDNA was then generated using the entire sample and TaqMan Reverse Transcription Reagents with random hexamers (Applied Biosystems). The qRT-PCR reactions were performed as described above, using 1 μl of cDNA per reaction. The primers used to detect transfected L1 RNAs were directed against the neomycin resistance cassette present in pJM101/L1.3 and pJM101/L1.3 3′UTRΔ (shown in Supplementary Figure S1). The sequences of the primers were *L1 NEO* FW: 5′-TCAGAAGAACTCGTCAAGAA-3′ and *L1 NEO* RV: 5′-CGGACCGCTATCAGGACATA-3′. Relative quantitation of RNAs was performed using the delta delta Ct method, as described above.

### siRNA transfection

Lipofectamine 2000 (Invitrogen) was used to transfect either Non-Targeting siRNA (40nM; D-001206-13-05, siGENOME Non-Targeting siRNA pool) or L1 siRNA directed against ORF1p (5′-GAAAUGAAGCGAGAAGGGAAGUUUA-3′) (12.5 nM; Dharmacon (66,67)) into HT-29 cells, according to the protocol provided by the manufacturer. Briefly, ∼8 × 10^4^ cells were plated in 6-well tissue culture plates and transfected the following day. Forty-eight hours following transfection, RNA and protein were harvested to assess knockdown efficiency. Of note, the knockdown efficiency of two additional L1 siRNAs directed against ORF1p were also tested (66,67) (5′-AAGAAATGAGCAAAGCCTCCAAGAA-3′, and 5′-TCAGCAATGGAAGATGAAATGAATG-3′), but these L1 siRNA were not used in subsequent experiments due to their inability to result in efficient knockdown in our cell lines.

### Proximity ligation assay (PLA) and quantification

The proximity ligation assay was performed using the DuoLink® PLA kit (Sigma Aldrich) according to the protocol provided by the manufacturer. An illustration of the protocol is available at https://www.sigmaaldrich.com/US/en/deepweb/assets/sigmaaldrich/marketing/global/documents/267/186/amplified-detection-duolink-pla.pdf. Cells were plated onto glass coverslips, transfected or treated with DMSO or NRTIs, and then fixed using an aqueous 4% formaldehyde solution in PBS for 10 minutes. Following fixation, the samples were permeabilized with 0.2% triton-X on ice for 5 min. All washes performed throughout the assay were done twice at room temperature using the previously prepared wash buffers supplied by Sigma. One drop of the Duolink® Blocking Solution was added to each sample and incubated in a pre-heated humidity chamber for 60 min at 37°C. Following blocking, the primary antibodies to NCAPD3 (anti-rabbit) or NCAPD2 (anti-mouse) were added to each sample and allowed to incubate overnight at 4°C. The next day, the primary antibody was removed, the coverslips were washed in supplied buffers, according to manufacturer's protocol, and the PLA probe solution was applied to each sample. The samples were incubated in a pre-heated humidity chamber for 60 minutes at 37°C. The PLA probe solution was removed and the samples were washed in supplied buffers, according to manufacturer's protocol. The ligation solution was applied according to manufacturer's protocol and the samples were incubated in a pre-heated humidity chamber for 30 min at 37°C.The ligated samples were washed in supplied buffers according to manufacturer's protocol and the amplification solution provided in the kit (containing DNA Polymerase) was applied according to manufacturer's protocol. The samples were again incubated in a pre-heated humidity chamber for 100 min at 37°C. Finally, the samples were washed in supplied buffers, according to manufacturer's protocol and then mounted to a glass slide using a one drop of Duolink® In Situ Mounting Medium with DAPI. At least three independent images were taken from different locations on a coverslip using a Leica SP8 DMI8 inverted confocal microscope (63× objective). One coverslip was considered to be an independent experiment; at least two independent experiments were performed for each condition tested. Nuclear PLA foci were identified and quantified from 3D images of individual focal planes within z-stacks, using Volocity 3D Image Analysis Software v 6.5.1 (Quorum Technologies Inc. Puslinch, Ontario). Compartmentalization analysis of the PLA foci within the 3D images allowed us to quantify foci that localized within the DAPI-stained nuclear compartment. The remaining PLA foci located outside of the nucleus were also quantified for each image. The total numbers of nuclear foci and cytoplasmic foci were divided by the total number of nuclei present in the image to obtain the average number of nuclear or cytoplasmic PLA foci/cell. Between 50 and 150 nuclei were evaluated per image.

### Cell cytotoxicity of reverse transcriptase (RT) inhibitors

Assays to monitor the potential cytotoxicity of RT inhibitors were performed as previously described (68). RT inhibitors were first made as 50 mM stock solutions in DMSO and then diluted in tissue culture media to arrive at the concentrations used in the respective experiments. Briefly, untransfected HT-29 cells were plated on 10 cm plates (500 cells/plate) with and without the RT inhibitors Didanosine and Zidovudine (Sigma Aldrich). Ten days later, cells were fixed to the plate and stained with 0.1% crystal violet using the same procedure described above for retrotransposition assays. The number of foci was counted to calculate colony formation ability and compared to cells treated with equal concentrations of DMSO alone.

### RT Inhibitors and L1 retrotransposition

The cell-based retrotransposition assay was conducted as described above. Seventy-two hours after transfection, the cells were trypsinized and plated onto three 10cm plates in RPMI medium containing 600 μg/ml G418 and 50 μM RT inhibitors Didanosine and Zidovudine (Sigma Aldrich). RPMI medium containing 600 μg/ml G418 and 50 μM DMSO was added to the mock treated plates. Two weeks post-transfection, G418 resistant cells were fixed to the plate and stained with 0.1% crystal violet, using the same procedure described above for retrotransposition assays, to calculate the retrotransposition frequency. Three independent assays were performed for each experiment.

### Statistical analyses

Data are expressed as mean ± SD (*n* = 3 independent experiments performed under identical conditions). Statistical analyses were performed using GraphPad Prism^©^ (GraphPad Software) with an unpaired Student's *t*-test. A *P*-value < 0.05 was considered statistically significant.

## RESULTS

### The L1 3′UTR is required for condensin II and GAIT association with L1 mRNA

We previously demonstrated that condensin II interacts with the GAIT complex to promote GAIT complexation, which, in turn, facilitates condensin II and GAIT association with L1 RNA (31). This association can inhibit binding of the translation initiation complex to the L1 RNA 5′UTR, allowing a modest repression of L1 ORF1p translation (31). We also hypothesized that a putative GAIT element, which is projected to form a stem-loop structure similar to those previously shown to be bound by GAIT in monocytes (69), is present within the L1 RNA 3′UTR (31).

**Figure 1.**
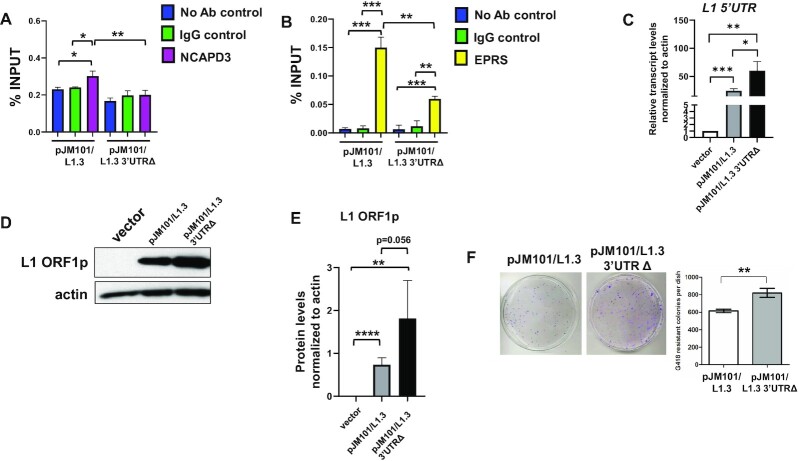
Condensin II and GAIT proteins associate with the 3′UTR of L1 RNA. **(A, B)** HT-29 cells were transfected with either a full-length L1 (pJM101/L1.3) or a L1 harboring a deletion within its 3′UTR (pJM101/L1.3 3′UTRΔ) and RIP/qRT-PCR assays were conducted using either a (A) NCAPD3 antibody, or (B) EPRS antibody to immunoprecipitate L1 RNA. Reactions lacking an antibody (No Antibody IP) and reactions involving a non-specific, IgG antibody served as negative controls. Sequences within the neomycin resistance cassette present in pJM101/L1.3 and pJM101/L1.3 3′UTRΔ were used to design qRT-PCR primers to detect L1 RNA. The average levels of L1 RNA-associated protein were calculated as percentages of the input used for each immunoprecipitation; averages from three independent experiments are shown. (**C**) L1 transcripts in HT-29 cells transfected with either the pCEP4 empty vector, pJM101/L1.3 or pJM101/L1.3 3′UTRΔ were analyzed by qRT-PCR. Transcripts were normalized to actin transcript levels and the averages of four independent experiments are shown. (**D**) Representative immunoblot analysis of L1 ORF1p levels in HT-29 cells transfected with either the pCEP4 empty vector, pJM101/L1.3 or pJM101/L1.3 3′UTRΔ. (**E**) Band intensities from four biological replicates of the experiment shown in panel C were quantified from film and normalized to actin. (**F**) Retrotransposition assays performed using HT-29 cells transfected with either pJM101/L1.3 or pJM101/L1.3 3′UTRΔ. Quantification (right) of three independent experiments. *P* values were determined by performing unpaired *t*-tests. *P ≤ 0.05, **P ≤ 0.01, ***P ≤ 0.001, ****P ≤ 0.0001. Error bars indicate standard deviations from the mean.

To test whether the L1 RNA 3′UTR contains sequences important for the interaction of L1 RNA with the NCAPD3/condensin II and EPRS/GAIT complexes, a HT-29 human colon adenocarcinoma epithelial cell line was transfected with engineered constructs expressing either a full-length L1 (pJM101/L1.3) or a L1 harboring a deletion of sequences within its 3′UTR (pJM101/L1.3 3′UTRΔ) (Supplementary Figure S1). RNA-immunoprecipitation (RNA-IP) followed by qRT-PCR was performed to determine if L1 RNAs were co-precipitated by the NCAPD3/condensin II and EPRS/GAIT complexes. Immunoblotting analyses demonstrated relatively equal amounts of immunoprecipitated NCAPD3 and EPRS protein in lysates from cells expressing pJM101/L1.3 and pJM101/L1.3 3′UTR Δ (Supplementary Figure S2). However, the ability of NCAPD3 and of EPRS to associate with L1 RNAs was significantly reduced in cells transfected with pJM101/L1.3 3′UTRΔ when compared to pJM101/L1.3 transfected cells, suggesting that sequences, or perhaps a structure(s), within the L1 3′UTR are important for condensin II and GAIT to associate with L1 RNAs (Figure 1A and B). Cells transfected with pJM101/L1.3 3′UTRΔ also revealed a modest (∼2-fold) increase in the steady state level of L1 RNA and L1 ORF1p (Figure 1C–E). Results of retrotransposition assays revealed an approximate 25% increase in L1 retrotransposition efficiency in HT-29 cells transfected with pJM101/L1.3 3′UTRΔ, when compared to cells transfected with pJM101/L1.3 (Figure 1F). Combined with our previous data (31), these findings suggest that condensin II and GAIT associate with the L1 RNA 3′UTR to repress binding of the eIF4F complex, leading to a reduction in ORF1p levels and L1 retrotransposition efficiency.

### Condensin I represses L1 retrotransposition in human cells

**Figure 2.**
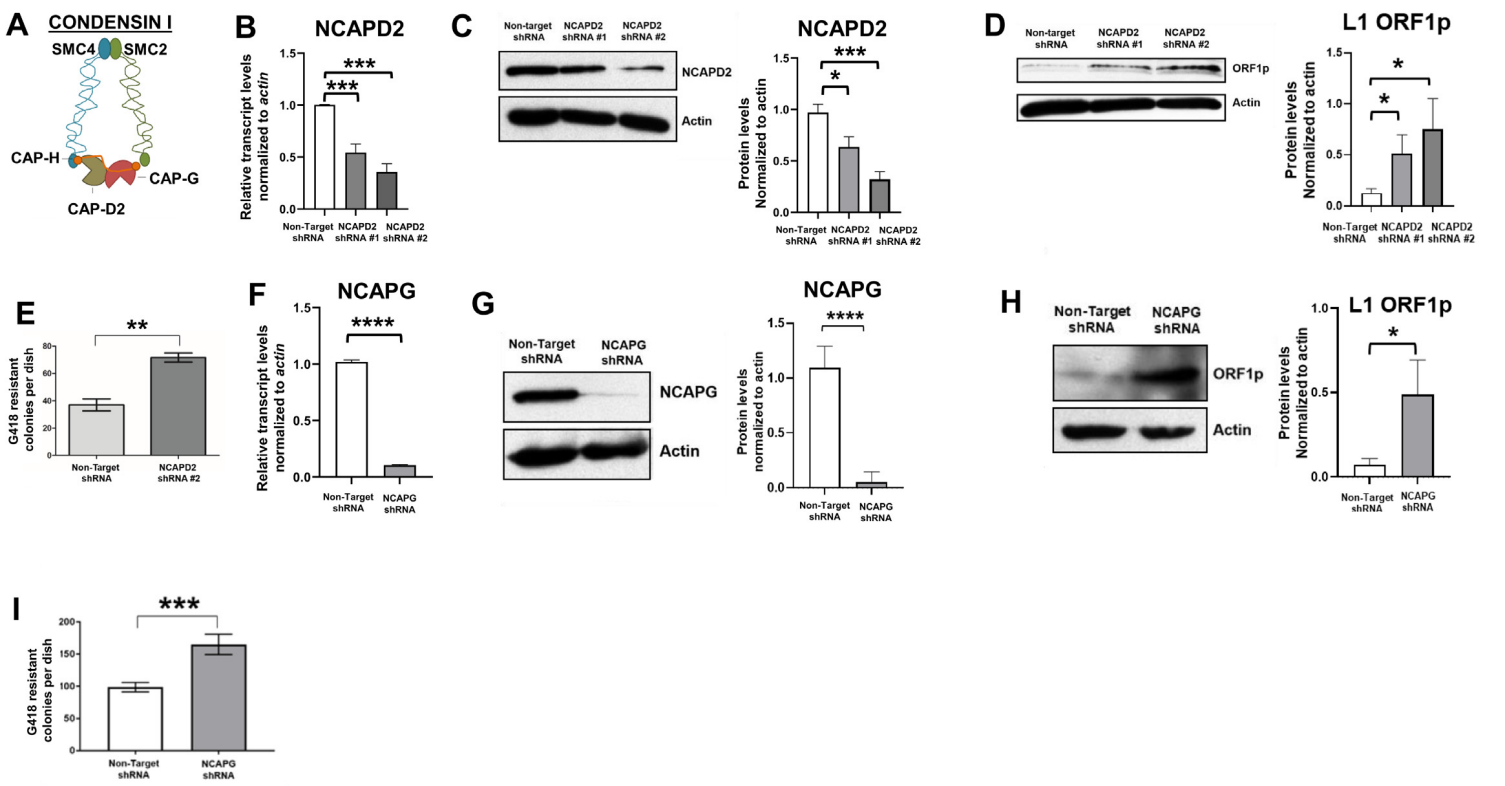
Condensin I represses L1 retrotransposition and expression. (**A**) Schematic of the condensin I complex; the names of condensin I subunits are indicated by the different colored shapes. (**B–D**) HT-29 cells were induced to express either Non-target, control shRNA or NCAPD2 shRNA. (B) Cellular RNAs were analyzed by qRT-PCR to detect *NCAPD2* transcript levels. Transcripts were normalized to *actin* transcript levels; the averages of three independent experiments are shown. Whole cell lysates were subjected to immunoblot analysis to detect (C) NCAPD2 protein or (D) endogenous ORF1p. Quantification of three independent experiments are shown to the right of each blot in panels C and D. Band intensities were quantified from film and normalized to actin. (**E**) HT-29 cells were induced to express either Non-Target shRNA or NCAPD2 shRNA and then were transfected with equal amounts of a pJM101/L1.3 expression vector to assess retrotransposition efficiency. Quantification of three independent experiments is shown. (**F–H**) HT-29 cells were induced to express either Non-target, control shRNA or NCAPG shRNA. (F) Cellular RNAs were analyzed by qRT-PCR to detect *NCAPG* transcript levels. Transcripts were normalized to *actin* transcript levels, and the averages of three independent experiments are shown. Whole cell lysates were subjected to immunoblot analysis to detect (G) NCAPG protein or (H) endogenous ORF1p. Quantification of three independent experiments is shown to the right of each blot in panels G and H. Band intensities were quantified from film and normalized to actin. (**I**) HT-29 cells were induced to express either Non-Target shRNA or NCAPG shRNA and then were transfected with equal amounts of pJM101/L1.3 to assess retrotransposition efficiency. Quantification of three independent experiments is shown. P values were determined by performing unpaired *t*-tests. *P ≤ 0.05, **P ≤ 0.01, ***P ≤ 0.001, ****P ≤ 0.0001. Error bars indicate standard deviations from the mean.

The ability of condensin II to repress L1 retrotransposition prompted us to ask whether the other condensin complex present in eukaryotic cells, condensin I, may also act to repress L1 retrotransposition. Thus, we generated HT-29 cells expressing inducible small hairpin RNAs (shRNA) targeting the condensin I subunits NCAPD2 (Sigma TRCN0000115682 as shRNA#1 and Sigma TRCN0000244475 as shRNA #2) and NCAPG (Sigma TRCN0000143554) (Figure 2A–C, F, G), as well as a control cell line expressing a control Non-Targeting (NT) shRNA (Sigma SHC001). Immunoblot analyses revealed that NCAPD2- and NCAPG-deficient cells expressed approximately 3- to 5-fold higher levels of endogenous L1 ORF1p and exhibited a modest increase (∼1.5- to 2-fold) in pJM101/L1.3 L1 retrotransposition efficiency (as demonstrated by results of retrotransposition efficiency assays) when compared to the NT shRNA control cell line (Figure 2D, E, H, I). The residual amounts of NCAPD2 and NCAPG protein present in the cells following inducible shRNA expression (Figure 2C and G) may account for the small but significant changes observed in our experiments. These data suggest that, like condensin II, condensin I also may restrict L1 retrotransposition. As controls, immunoblotting analyses further revealed that the depletion of neither NCAPD2 (Supplementary Figure S3) nor NCAPG (Supplementary Figure S4) significantly affected condensin II protein levels.

### Condensin I promotes condensin II and GAIT protein association with L1 mRNA

**Figure 3.**
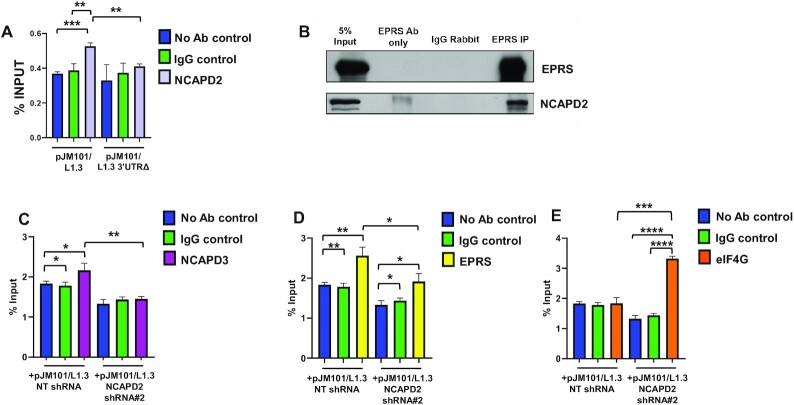
NCAPD2/ condensin I is required for EPRS and NCAPD3 association with L1 RNA. (**A**) HT-29 cells were transfected with either pJM101/L1.3 expression vector or a L1 construct harboring a deletion within its 3′UTR (pJM101/L1.3 3′UTRΔ) and RIP/qRT-PCR assays were conducted using a NCAPD2 antibody to immunoprecipitate L1 RNA. Reactions lacking an antibody (No Antibody IP) and reactions involving a non-specific, IgG antibody served as negative controls. Sequences within the neomycin resistance cassette present in pJM101/L1.3 and pJM101/L1.3 3′UTRΔ were used to design qRT-PCR primers to detect L1 RNA. The average levels of RNA-bound protein were calculated as percentages of the input used for each immunoprecipitation; averages from three independent experiments are shown. (**B**) EPRS co-IP/immunoblot experiments were conducted in HT-29 cells to detect association with NCAPD2. IPs using antibody only (ie. no lysate) and IPs using an IgG antibody served as negative controls. A representative blot of three independent experiments is shown. (**C–E**) HT-29 cells were induced to express either NT shRNA or NCAPD2 shRNA and then were transfected with pJM101/L1.3. RIP/qRT-PCR assays were conducted using either (C) NCAPD3 antibody, (D) EPRS antibody, or (E) eIF4G antibody to immunoprecipitate L1 RNA, as described for (A). *P* values were determined by performing unpaired *t*-tests. *P ≤ 0.05, **P ≤ 0.01, ***P ≤ 0.001, ****P ≤ 0.0001. Error bars indicate standard deviations from the mean.

We next conducted RNA-IP/qRT-PCR experiments in HT-29 cells transfected with pJM101/L1.3 or pJM101/L1.3 3′UTRΔ to determine whether condensin I protein, NCAPD2, associates with L1 RNA. Immunoprecipitation of NCAPD2 protein, followed by qRT-PCR analyses to detect co-precipitated L1 RNAs, revealed that NCAPD2 significantly associated with full length L1 RNA when compared to negative control RNA-IP reactions without an antibody or with IgG antibody (Figure 3A). Moreover, although equal amounts of NCAPD2 protein were immunoprecipitated from both populations of transfected cells (Supplementary Figure S5), the association of NCAPD2 was diminished in cells transfected with pJM101/L1.3 3′UTRΔ (Figure 3A), suggesting the L1 RNA 3′UTR is important for the association of NCAPD2 and L1 RNA.

We next tested whether NCAPD2 could behave similarly to condensin II proteins and associate with EPRS protein. Indeed, immunoprecipitation of EPRS co-precipitated NCAPD2 protein in HT-29 cells (Figure 3B). To test whether NCAPD2 was required for the association of NCAPD3 and EPRS proteins with L1 RNA, we inducibly knocked down NCAPD2 in HT-29 cells and performed RNA-IP/qRT-PCR analyses. Interestingly, depleting NCAPD2 led to a drastic reduction of EPRS and NCAPD3 association with L1 RNA (Figure 3C and D). Control co-IP experiments further revealed that NCAPD2 depletion did not affect the association of the NCAPD3 and EPRS proteins (Supplementary Figure S6). Moreover, RNA-IP/qRT-PCR experiments revealed that NCAPD2 depletion led to a ∼1.5-fold increase in eIF4G binding to L1 RNAs (Figure 3E), which is similar to previous data reported in NCAPD3 depleted cells (31). Control immunoblotting experiments demonstrated that equal amounts of NCAPD3 and EPRS protein were immunoprecipitated in cells expressing Non-target shRNA as compared to cells expressing NCAPD2 shRNA (Supplementary Figures S7A, S7B). However, slightly lower amounts of eIF4G protein were immunoprecipitated from cells expressing NCAPD2 shRNA, suggesting that we may actually be underestimating the observed increases in eIF4G-associated L1 RNA in NCAPD2 depleted cells (Supplementary Figure S7C). These data further support the hypothesis that NCAPD2/condensin I may play a role in NCAPD3/condensin II and GAIT-mediated repression of L1 mRNA translation.

### Condensin I and II proteins form a Super Condensin Complex (SCC) in an L1-dependent manner

**Figure 4.**
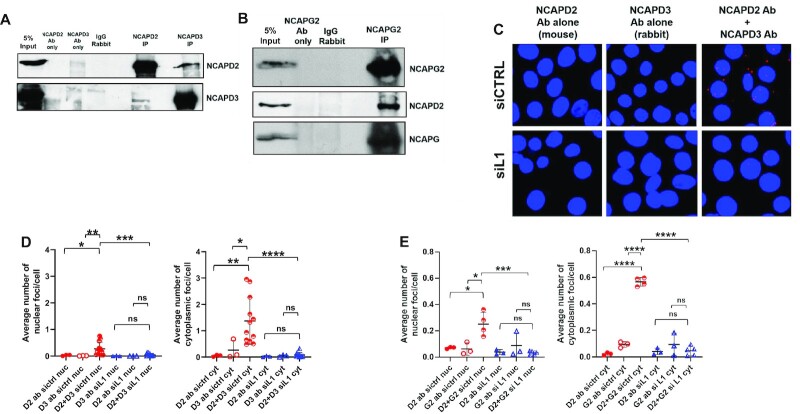
NCAPD2 and NCAPD3 associate to form SCCs in a L1 RNA dependent manner. (**A**) Reciprocal NCAPD3 and NCAPD2 co-IP/immunoblots were conducted in HT-29 cells to detect an association between NCAPD3 and NCAPD2. Antibody only (i.e. no lysate) and IgG antibody IPs served as negative controls. Each experiment was performed three times and representative blots are shown. (**B**) NCAPG2 co-IP/immunoblot experiments were conducted in HT-29 cells to detect an association with NCAPD2 (middle panel) and with NCAPG (bottom panel). Antibody only (i.e. no lysate) and IgG antibody IPs served as negative controls. Each experiment was performed three times and representative blots are shown. (**C**) Proximity Ligation Assays (PLAs) were performed to detect an association between NCAPD2 and NCAPD3 in HT-29 cells transfected with control (siCTRL) or L1 siRNA (siL1). Single antibody controls were performed in parallel for each experiment. Images were taken using a confocal microscope with a 63x objective; maximum projections are shown. (**D**) Volocity imaging software was used to analyze confocal images and quantify the average number of nuclear and cytoplasmic PLA foci per cell. Each dot represents the average of 50–150 nuclei evaluated from a single image. Images were taken from three independent experiments. (**E**) PLAs were performed to detect associations between NCAPD3 and NCAPG2 in HT-29 cells transfected with siCTRL or siL1 and results were quantified as described in (D). *P* values were determined by performing unpaired *t*-tests. *P ≤ 0.05, **P ≤ 0.01, ***P ≤ 0.001, ****P ≤ 0.0001, ns = not significant. Error bars indicate standard deviations from the mean.

Because condensin I and condensin II modestly repress L1 retrotransposition and NCAPD2 promotes condensin II protein association with L1 mRNA in HT-29 cells, we next tested whether condensin I and condensin II can interact. Reciprocal co-IP/immunoblot experiments demonstrated an association between NCAPD2 and NCAPD3 in whole cell lysates prepared from HT-29 cells (Figure 4A). A similar result also was observed with other condensin I and condensin II subunits (Figure 4B and Supplementary Figure S8). Thus, we termed the complexes containing condensin I and II proteins, Super Condensin complexes (SCCs).

To examine whether the association between condensin I and condensin II subunits required the presence of endogenous L1 RNA and/or L1 proteins, we tested whether the transfection of HT-29 cells with a siRNA directed against L1 ORF1p affected SCC formation (see Methods). Transfection of the L1 siRNA led to a ∼2-fold decrease in both L1 RNA and L1 ORF1p levels in cells (Supplementary Figures S9A-S9C), but did not significantly affect NCAPD3 or NCAPD2 protein levels (Supplementary Figures S9D, E). Subsequent Proximity Ligation Assays (PLA) in HT-29 cells, which allow the *in situ* detection of endogenous proteins in close proximity to one another (see Methods), revealed that SCC formation primarily occurred in the cytoplasm and that L1 siRNA transfection decreased SCC formation by approximately 3-fold in both the nucleus and cytoplasm when compared to control siRNA treated cells (Figure 4C–E).

**Figure 5.**
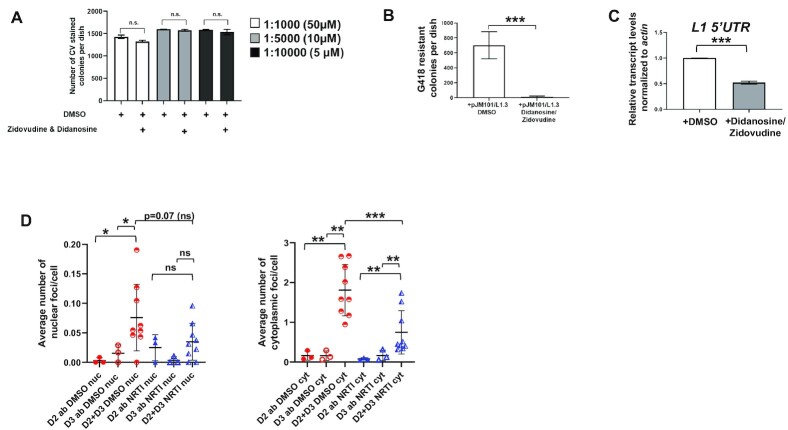
NRTI Treatment reduces L1 transcripts and L1 retrotransposition events and decreases cytoplasmic SCC formation. (**A**) HT-29 cells were treated with either DMSO or DMSO containing Zidovudine and Didanosine at the indicated drug concentrations for 10 days then fixed and stained with crystal violet (CV) to measure cell toxicity. Shown are averages from three independent experiments. (**B**) Retrotransposition assays were performed using HT-29 cells that were transfected with pJM101/L1.3 and treated with either DMSO or a combination of the nucleoside reverse transcriptase inhibitors (NRTIs) Zidovudine and Didanosine; each drug was at a final concentration of 50 μM in tissue culture media. Quantification of retrotransposition assays from three independent experiments is shown. (**C**) qRT-PCR analysis of endogenous L1 RNA levels (using a 5′UTR primer pair; See Supplementary Figure S1 and Methods) in HT-29 cells treated with either DMSO or NRTIs. The average relative transcript levels for three independent experiments are shown. (**D**) PLA was performed to detect an association between NCAPD2 and NCAPD3 in HT-29 cells treated with DMSO or NRTIs at a final concentration of 50μM in tissue culture media. Single antibody controls were performed in parallel for each experiment. Images were taken using a confocal microscope with a 63x objective and maximum projections are shown. Volocity imaging software was used to analyze confocal images as noted in Figure 4 to quantify the average number of nuclear (left chart) and cytoplasmic (right chart) PLA foci per cell. *P* values were determined by performing unpaired *t*-tests. *P ≤ 0.05, **P ≤ 0.01, ***P ≤ 0.001, ns = not significant. Error bars indicate standard deviations from the mean.

L1 retrotransposition requires L1 ORF2p reverse transcriptase activity (35,70) and the treatment of transfected cells with Nucleoside Reverse Transcriptase Inhibitors (NRTIs) severely inhibits the retrotransposition of engineered L1s in cultured cells (68,71,72). To examine whether L1 retrotransposition efficiency correlated with SCC formation, we treated HT-29 cells with a combination of the NRTIs Didanosine and Zidovudine. Didanosine and Zidovudine have been shown to inhibit retrotransposition in different organisms and different cell types (73,74); it also should be noted that these NRTIs also inhibit telomerase (75,76). Control experiments demonstrated that Didanosine and Zidovudine reduced L1 retrotransposition by greater than 2-orders-of-magnitude, but did not significantly affect HT-29 cell viability (Figure 5A, B). Notably, qRT-PCR experiments, using primers directed against sequences in the L1 5′UTR, revealed that Didanosine/Zidovudine treatment led to a 2-fold reduction in the steady state levels of endogenous L1 transcripts in cells (Figure 5C). Intriguingly, PLA assays performed in the Didanosine/Zidovudine treated cells revealed a ∼2-fold reduction in cytoplasmic SCC formation (Figure 5D), similar to results observed in cells treated with L1 siRNA, further supporting the notion that endogenous L1 RNA abundance, but not RT-activity or retrotransposition, *per se*, may play a role in SCC formation in HT-29 cells.

### L1 expression promotes SCC formation in primary cells

**Figure 6.**
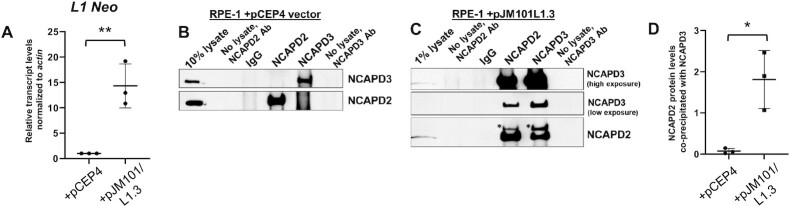
SCC formation occurs in primary cells expressing L1 transcripts. (**A**) qRT-PCR analysis of L1 transcripts in RPE-1 cells transfected with the pCEP4 empty vector or pJM101/L1.3 expression vector. Sequences within the neomycin resistance cassette present in pJM101/L1.3 were used to design qRT-PCR primers to detect L1 RNA. Transcript levels were normalized to *actin* and L1 transcript levels in pCEP4 transfected cells were set to 1. Shown is the average of three independent experiments. (**B, C**) IP/immunoblotting experiments for NCAPD2 and NCAPD3 were performed in RPE-1 cells transfected with (B) pCEP4 or (C) pJM101/L1.3. Antibody only (no lysate) and IgG antibody IPs served as controls. Asterisks indicate bands remaining after stripping the membrane that was immunoblotted for NCAPD3. Shown are representative blots of three independent experiments. (**D**) Average amounts of co-precipitated NCAPD2 protein, normalized to the amount of immunoprecipitated NCAPD3 protein, were quantified from the experiments in panels B and C. *P* values were determined by performing unpaired *t*-tests. *P ≤ 0.05, **P ≤ 0.01. Error bars indicate standard deviations from the mean.

To analyze whether SCC formation also occurred in primary cells, we transfected human RPE-1 cells, which are a hTERT-immortalized, non-transformed, primary cell line, with the pCEP4 empty vector or the pJM101/L1.3 expression vector (Figure 6A–D). Quantitative RT-PCR demonstrated significant increases in L1 transcripts in pJM101/L1.3 transfected cells when compared to the pCEP4 transfected control (Figure 6A). An association between NCAPD2 and NCAPD3 was not observed in pCEP4 transfected RPE-1 cells (Figure 6B), but a detectable NCAPD2/NCAPD3 association was observed in pJM101/L1.3 transfected RPE-1 cells (Figure 6C, D), suggesting that SCC formation can occur in primary cells and may also be dependent on L1 expression, as observed in HT-29 colon adenocarcinoma cells.

### SCC formation does not require condensin protein association with the L1 3′UTR

**Figure 7.**
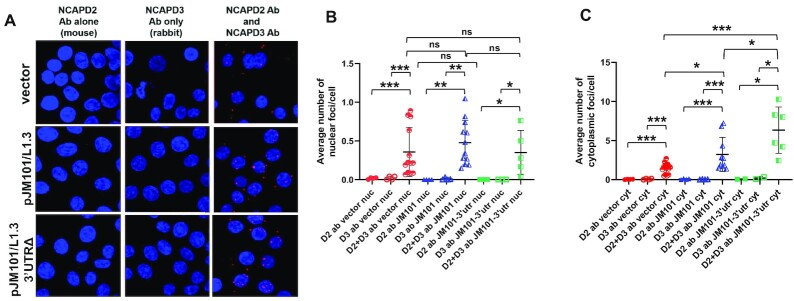
The 3′UTR of L1 RNA antagonizes SCC formation. (**A**) HT-29 cells were transfected with the pCEP4 empty vector, pJM101/L1.3 expression vector, or L1 expression vector harboring a 3′UTR deletion (pJM101/L1.3 3′UTRΔ) and Proximity Ligation Assays were performed to detect association between NCAPD2 and NCAPD3. Single antibody controls were performed in parallel for each experiment. Images shown were taken on a confocal microscope with a 63x objective and maximum projections are shown. (**B**, **C**) Volocity imaging software was used to analyze confocal images as noted in Figure 4D to quantify the average number of nuclear (B) and cytoplasmic (C) PLA foci per cell. Images shown were taken from two independent experiments. *P* values were determined by performing unpaired *t*-tests. *P ≤ 0.05, **P ≤ 0.01, ***P ≤ 0.001, ns = not significant. Error bars indicate standard deviations from the mean.

Sequences within the L1 3′UTR are necessary for the association of both NCAPD2 and NCAPD3 with L1 RNA (Figure 1). To examine whether the ability of NCAPD2 and NCAPD3 to associate with L1 RNA was important for SCC formation, we conducted PLA assays in HT-29 cells transfected with either pJM101/L1.3 or pJM101/L1.3 3′UTRΔ (Figure 7A-C). A ∼1.6-fold increase in cytoplasmic SCCs was observed in cells transfected with pJM101/L1.3 when compared to cells transfected with the pCEP4 empty vector (Figure 7C, compare blue half-open triangles to red half-open circles). By comparison, a ∼4.2-fold increase in cytoplasmic SCCs was observed in cells transfected with pJM101/L1.3 3′UTRΔ when compared to pCEP4 empty vector transfected cells (Figure 7C, compare green half-open squares to red half-open circles). This is equal to an approximate 2.6 fold increase in cytoplasmic SCCs in pJM101/L1.3 3′UTRΔ transfected cells when directly compared to pJM101/L1.3 transfected cells. Additional controls revealed that these differences were not due to changes in NCAPD2 or NCAPD3 protein levels in pJM101/L1.3 *vs*. pJM101/L1.3 3′UTRΔ transfected cells (Supplementary Figure S10), suggesting SCC formation in HT-29 cells does not require the L1 RNA 3′UTR. Instead, sequences within the L1 RNA 3′UTR actually may antagonize SCC formation.

## DISCUSSION

Collectively, our results suggest: (i) L1 expression promotes interactions between condensin I and condensin II to form SCCs which are primarily located within the cytoplasm of human epithelial cells; (ii) condensin I and condensin II proteins can associate with GAIT complex proteins and the L1 RNA 3′UTR; (iii) the condensin I subunit, NCAPD2 promotes the association of condensin II and GAIT proteins to RNA sequences and/or structures within the L1 3′UTR and (iv) the condensin I and condensin II proteins can repress L1 ORF1p expression and L1 retrotransposition.

**Figure 8.**
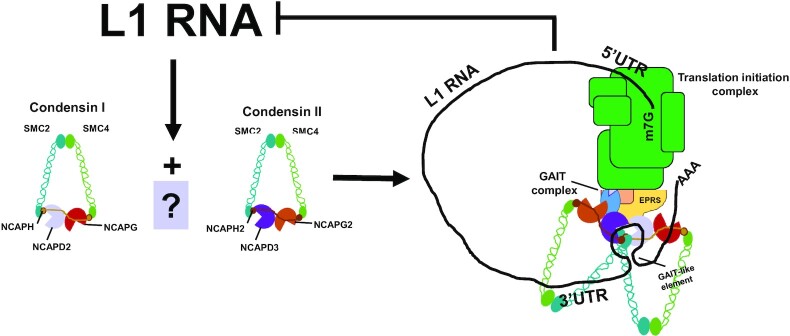
Working model for how condensin I and condensin II may affect L1 expression and retrotransposition. Condensin I and condensin II can primarily form cytoplasmic SCC complexes in a L1-dependent manner in both transformed and non-transformed human epithelial cells. We propose that condensins, either independently or following formation of the SCC, associate with GAIT complex proteins and the L1 RNA 3′ UTR to block translation of L1 mRNA (31) which subsequently leads to repression of L1 retrotransposition and prevents further increases in L1 RNA.

The above findings suggest a working model where L1 RNA expression stimulates the SCC formation in a L1 RNA dependent, but L1 RNA 3′UTR independent manner. After its formation, the SCC complex, through the actions of condensin I and condensin II complexes, then can associate with RNA sequences and/or structures within the L1 3′UTR to repress L1 ORF1p translation and L1 retrotransposition (Figure 8).

### Interactions between the L1 3′UTR and condensin proteins

The L1 3′UTR, which contains a putative GAIT element (31), is required for condensin I, condensin II and GAIT proteins to interact with L1 RNA (Figures 1 and Figure 3) and the deletion of sequences within the L1 3′UTR modestly increased L1 ORF1p expression and L1 retrotransposition (Figure 1). These data support our previous findings that condensin II and GAIT association with L1 RNA represses L1 ORF1p translation and retrotransposition (31). The GAIT complex originally was identified as an inhibitor of inflammatory mRNA translation in monocytes, in response to IFNγ-mediated signaling (55,56,77). Recent studies revealed that L1 expression induces IFN expression (73,78–83) and the subsequent transcription of Interferon Stimulated Genes, ultimately leading to L1 repression (73,80). Thus, it is tempting to speculate that the association of the condensin/GAIT complexes with the L1 RNA 3′UTR may yield insight into a wider array of inflammatory signaling pathways that serve to modulate L1 expression.

Our data further reveal that L1 expression stimulates SCC formation, but that SCC formation does not strictly require sequences within the L1 RNA 3′UTR (Figures 4 and 5). In contrast, the ectopic overexpression of pJM101/L1.3 3′UTRΔ actually led to a ∼4.2-fold increase in cytoplasmic SCCs when compared to an empty vector control and a ∼2.6-fold increase in cytoplasmic SCCs when compared to pJM101/L1.3 transfected cells (Figure 7). What could account for these findings? Building on our working model, we hypothesize that the deletion of sequences within the L1 RNA 3′UTR may allow L1 RNA to evade repression by condensins, which, in turn, results in a steady state increase in L1 RNA levels (Figure 1C) that can further stimulate SCC formation. Alternatively, it is possible that sequences within the L1 RNA 3′UTR lead to the activation of signaling pathways that repress SCC formation; thus deletion of this region could possibly lead to increased SCC formation. Regardless of which explanation (if any) is correct, future studies involving condensin protein mutants will be necessary to determine whether SCC formation is required for the ability of condensins to bind to the L1 3′UTR.

Notably, previous studies revealed that although the L1 3′UTR is not strictly required for retrotransposition (35), it contains an evolutionarily conserved guanine-rich homopurine tract, which is shared among other mammalian L1s, which, in rat L1s, is capable of forming triplex structures (84,85). Indeed, it will be interesting to determine the exact sequences and/or structures within L1 RNAs that bind condensin II and GAIT proteins in future studies.

### Condensin proteins cooperate in the cytoplasm to repress L1

Condensin I and condensin II generally are thought to be independent protein complexes that play roles in similar, yet distinct, cellular functions (2,3,86). Previous immunofluorescence analyses demonstrated that condensin I and condensin II localize to different chromosomal regions (2,3). However, condensin I and condensin II can also interact with the same chromosomal loci to regulate chromatin looping, chromatin accessibility, and gene expression in response to Estrogen Receptor α signaling (87). Our data reveals L1 expression promotes SCC formation, primarily in the cytoplasm (but also in the nucleus (see Figures 4 and 5), consistent with the finding that higher levels of condensin I reside in the cytoplasm during interphase (2,3,86). Although we observed SCCs in both dividing and non-dividing cells, most of the cells examined were not visibly in mitosis. Clearly, a more detailed analysis of the baseline levels of SCCs induced by endogenous L1 expression during each phase of the cell cycle would be required to determine SCC dynamics during cell cycle progression.

Whether SCCs form in the free cytosolic space or in association with specific cytoplasmic organelles requires further elucidation. Our laboratory recently demonstrated that condensin subunits localize to the outer membrane of mitochondria and within mitochondrial matrices and that condensin II regulates mitochondrial respiration and prevents reactive oxygen species (ROS) accumulation in response to stress (88). A previous report demonstrated that the repression of L1 RNA expression by the HUSH complex is involved in the MAVS-dependent activation of IFNβ in human cells (78). Interestingly, MAVS is located on the outer mitochondrial membrane, which is the same place where NCAPD3 and other condensin proteins localize (88). Thus, future experiments will be required to address whether the SCC forms on the outer mitochondrial membrane in response to L1 RNA expression and whether this subcellular localization is necessary for the association of the L1 RNA 3′UTR with condensins and GAIT/EPRS proteins. Finally, additional studies are needed to conclusively determine whether L1 RNA, L1 encoded proteins, or a byproduct of L1-specific enzymatic activity induces SCC formation.

The consequences of SCC formation in cellular homeostasis, if any, also requires elucidation. We only observed a small number of SCCs (approximately 1–2 foci per cell; Figure 4D) in response to increases in endogenous L1 expression and the numbers of SCC foci doubled in response to transfection with an active wild-type L1 (pJM101/L1.3). However, because we do not know the exact protein composition of SCC foci, whether the small numbers of SCCs observed in our studies have a dramatic impact on condensin protein nuclear functions, remains unknown; future ChIP-seq experiments will be required to formally assess any impacts.

### Might L1 expression play a role in condensin-deficient disease states?

Mutations in genes encoding condensin I and condensin II subunits are associated with various disease states. For example, biallelic mutations in NCAPD2, NCAPH, and NCAPD3 cause microcephaly (89). Interestingly, biallelic EPRS mutations have also been identified in patients with developmental disorders including microcephaly (90). However, direct connections between L1 and the development of microcephaly have not been reported. Finally, a recent HotNet2 Pan-Cancer analysis suggested that multiple cancers harbor rare mutations in SMC2, SMC4, NCAPD2, NCAPD3, NCAPH2 and NCAPG2, supporting a tumor-suppressor role for condensin proteins (26). Whether these mutations affect SCC formation or result in increases in L1 expression requires further exploration.

## MATERIALS AND CORRESPONDENCE

Further information and requests for reagents can be directed to and will be fulfilled by the corresponding author Michelle S. Longworth (longwom@ccf.org), Department of Inflammation and Immunity, Lerner Research Institute, Cleveland Clinic.

## Supplementary Material

gkac802_Supplemental_File
