# Supplementary material for: Condensin I and condensin II proteins form a LINE-1 dependent super condensin complex and cooperate to repress LINE-1
Source: Nucleic Acids Res. 2022 Sep 28;50(18):10680–94. doi: 10.1093/nar/gkac802 (PMC9561375; doi:10.1093/nar/gkac802)
Supplement: gkac802_Supplemental_File [file gkac802_supplemental_file.pdf]

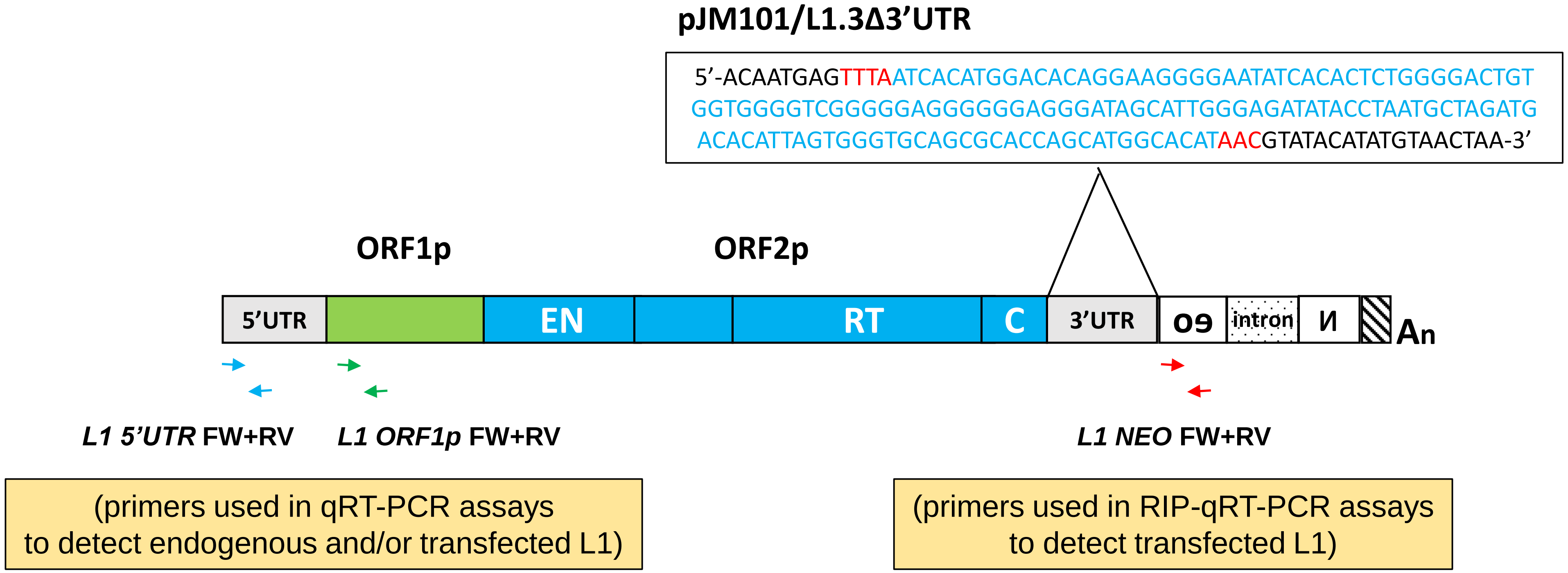

Ward Supp Fig. 1

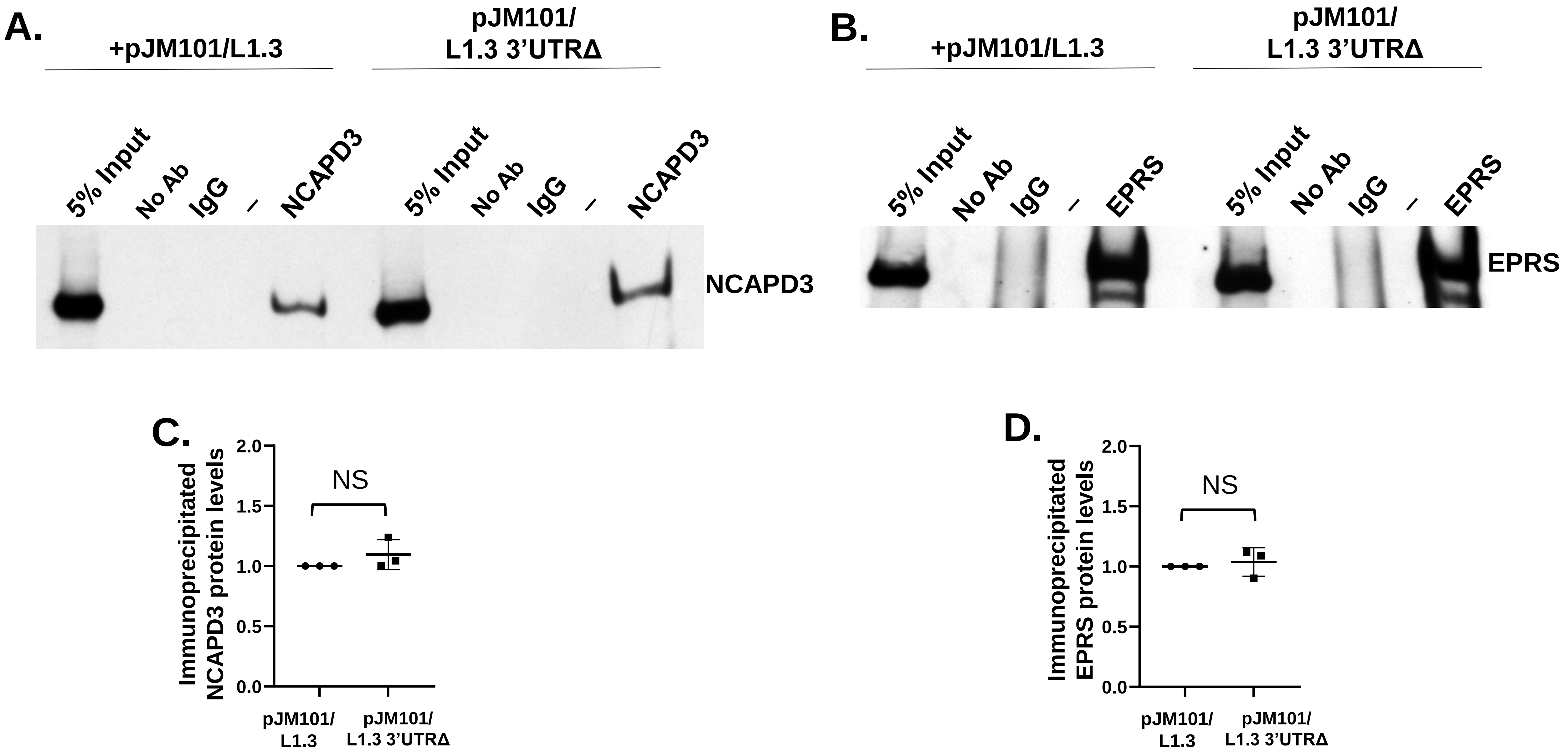

Ward Supp Fig. 2

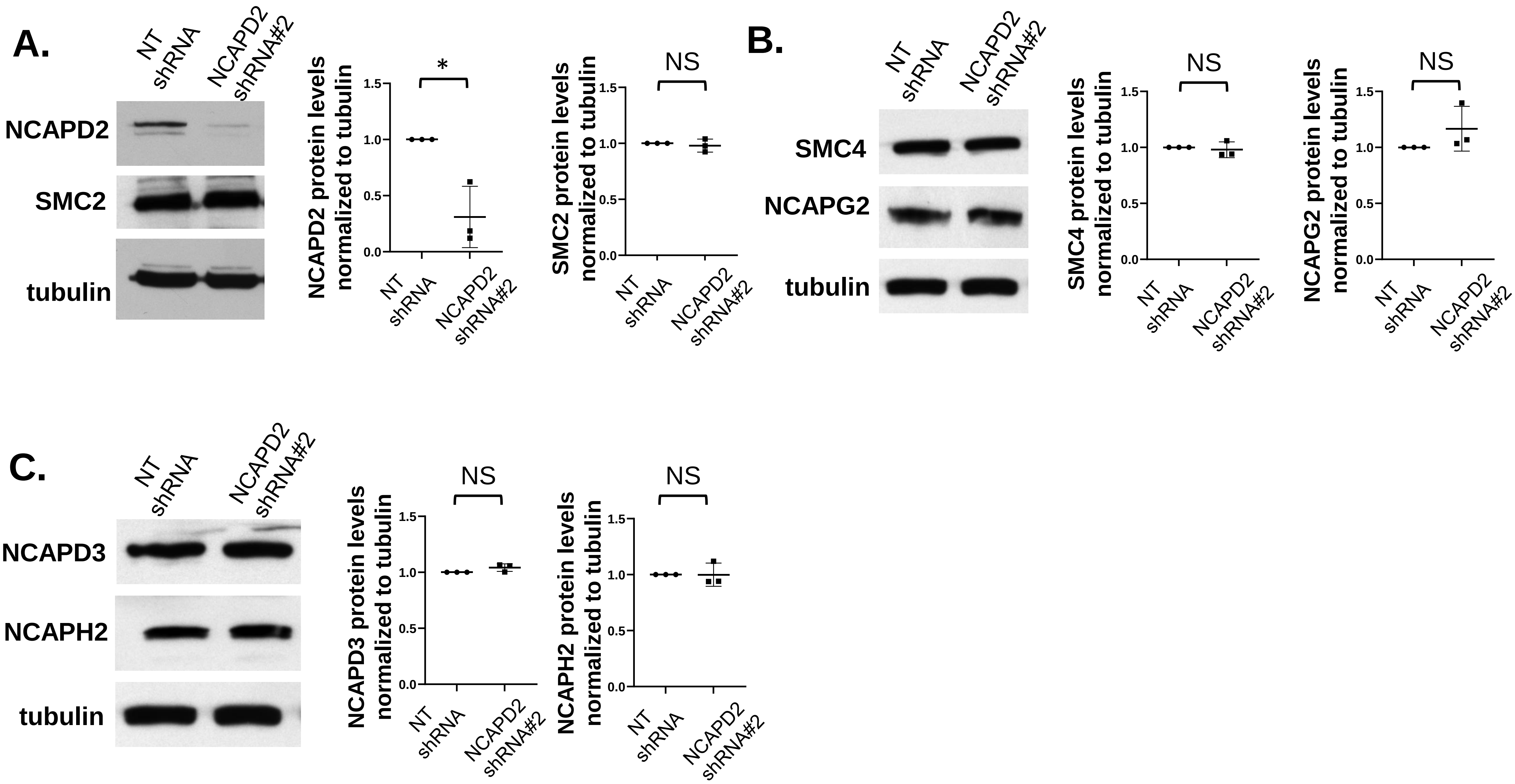

Ward Supp Fig. 3

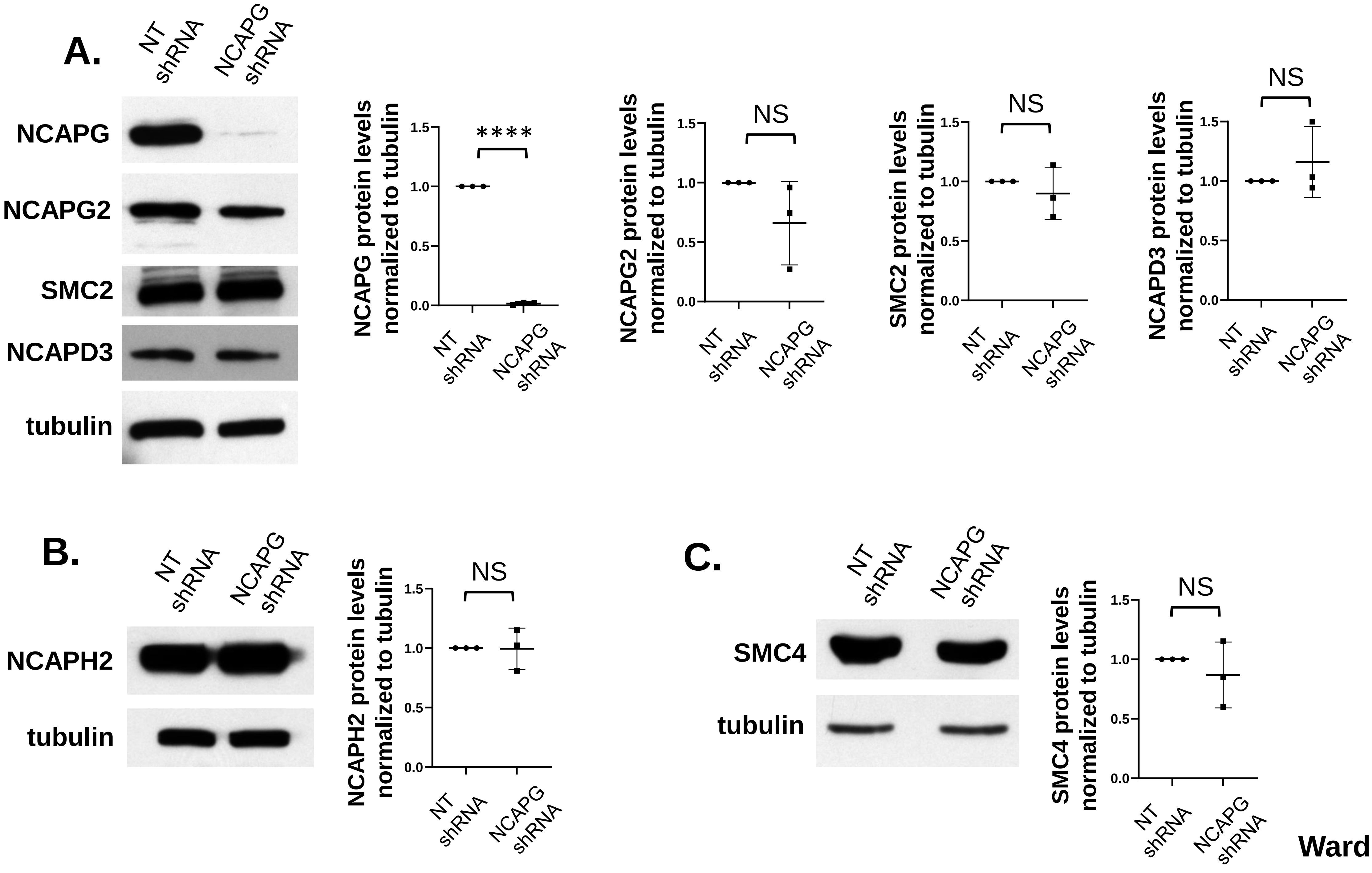

Ward Supp Fig. 4

A.

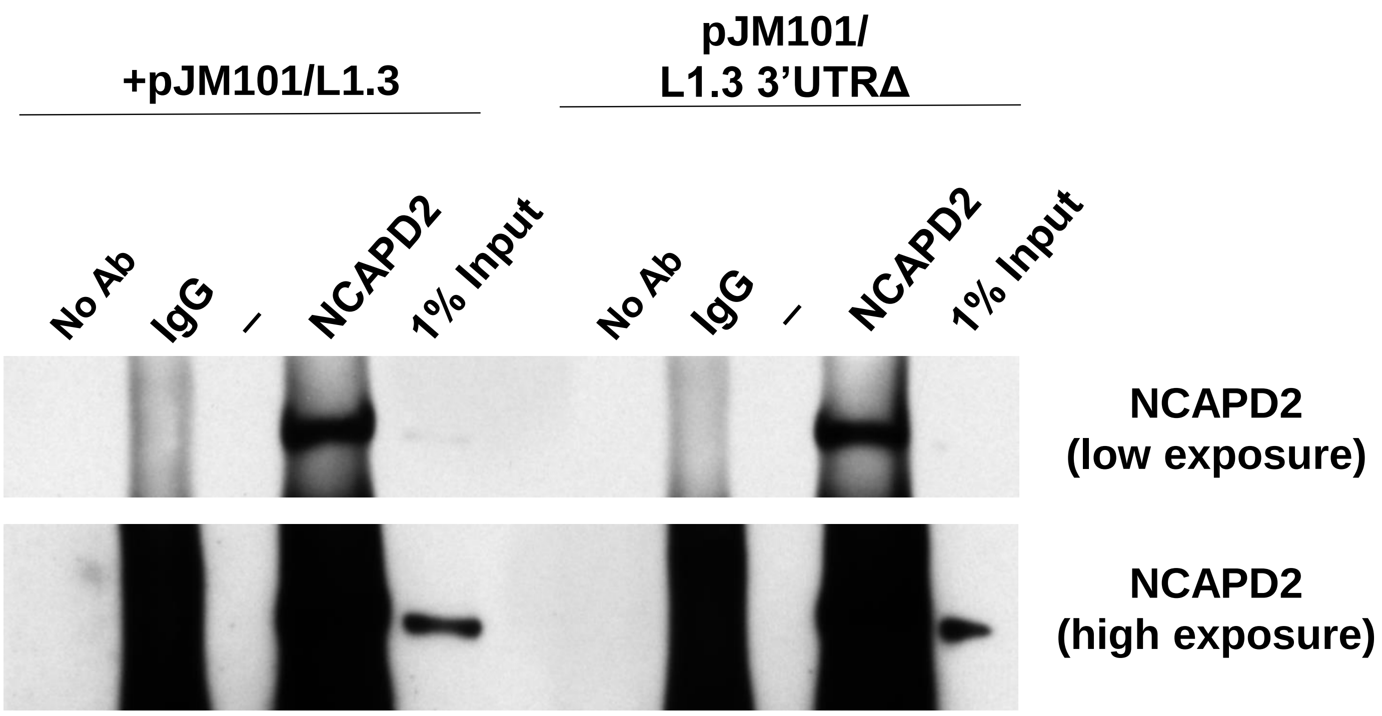

B.

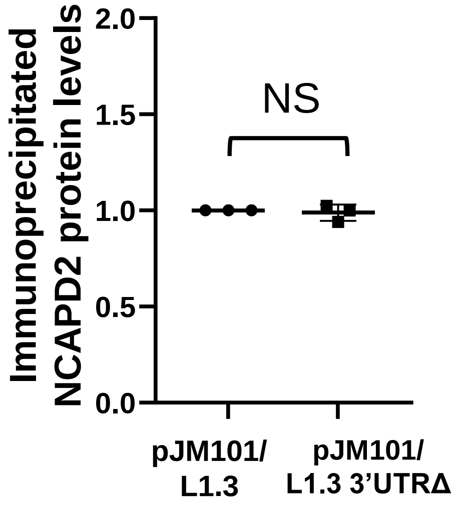

Ward Supp Fig. 5

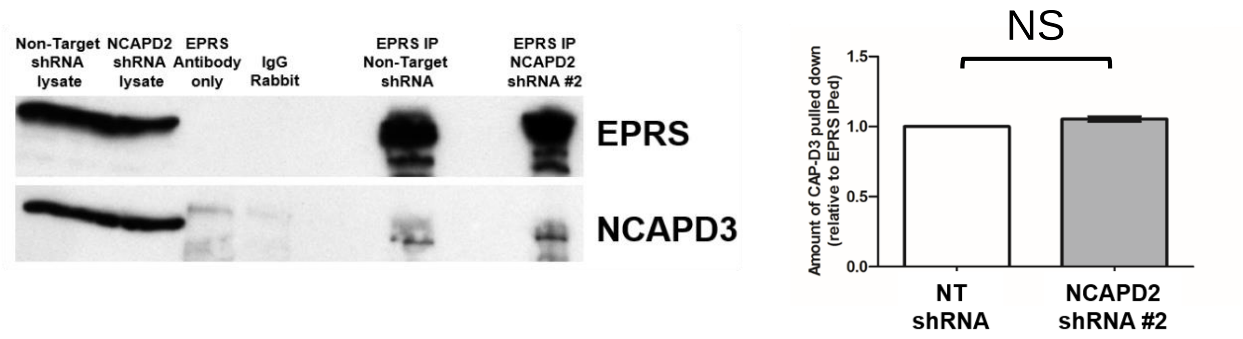

Ward Supp Fig. 6

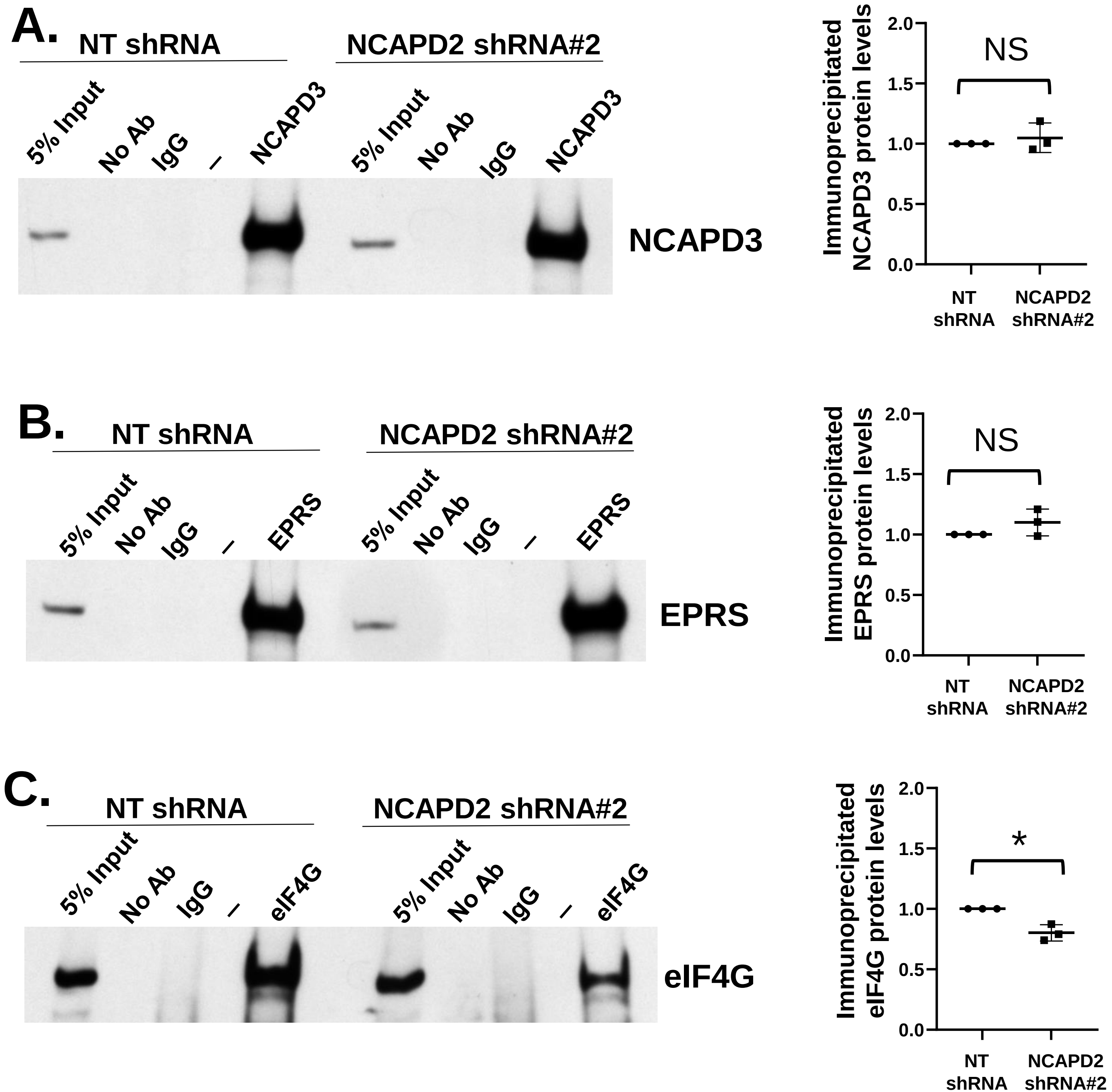

Ward Supp Fig. 7

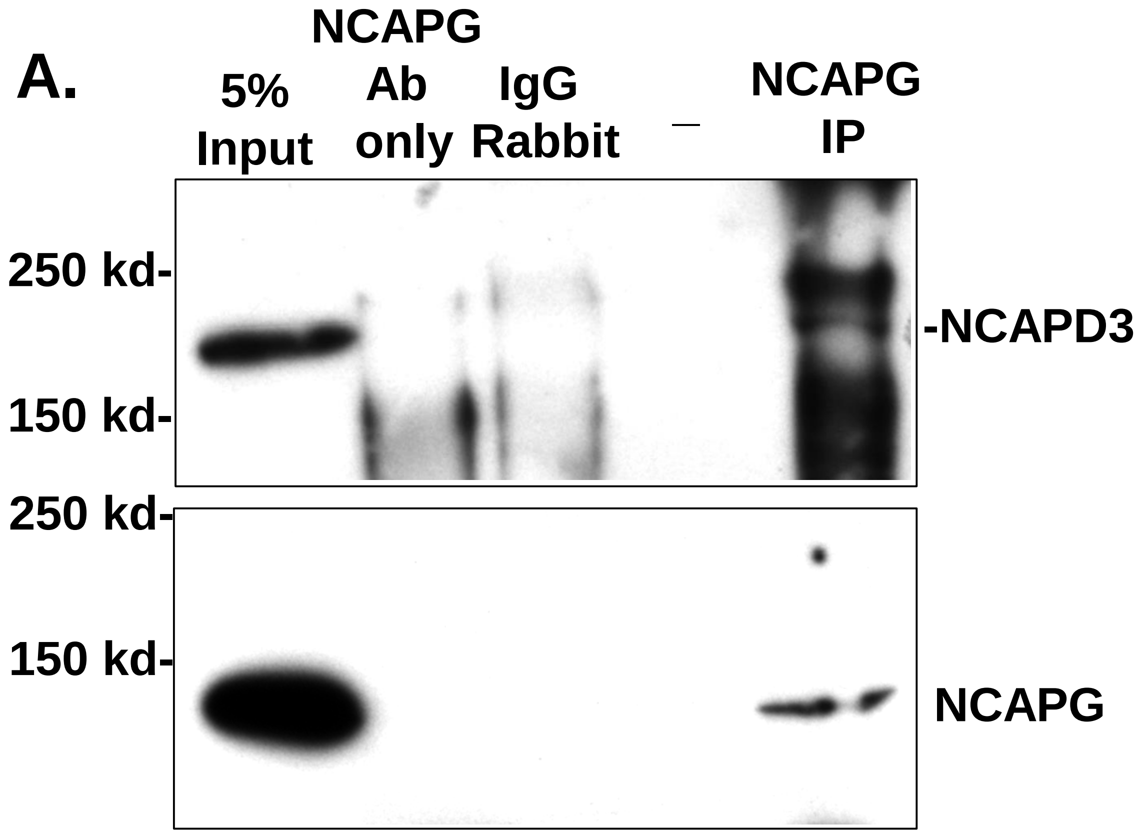

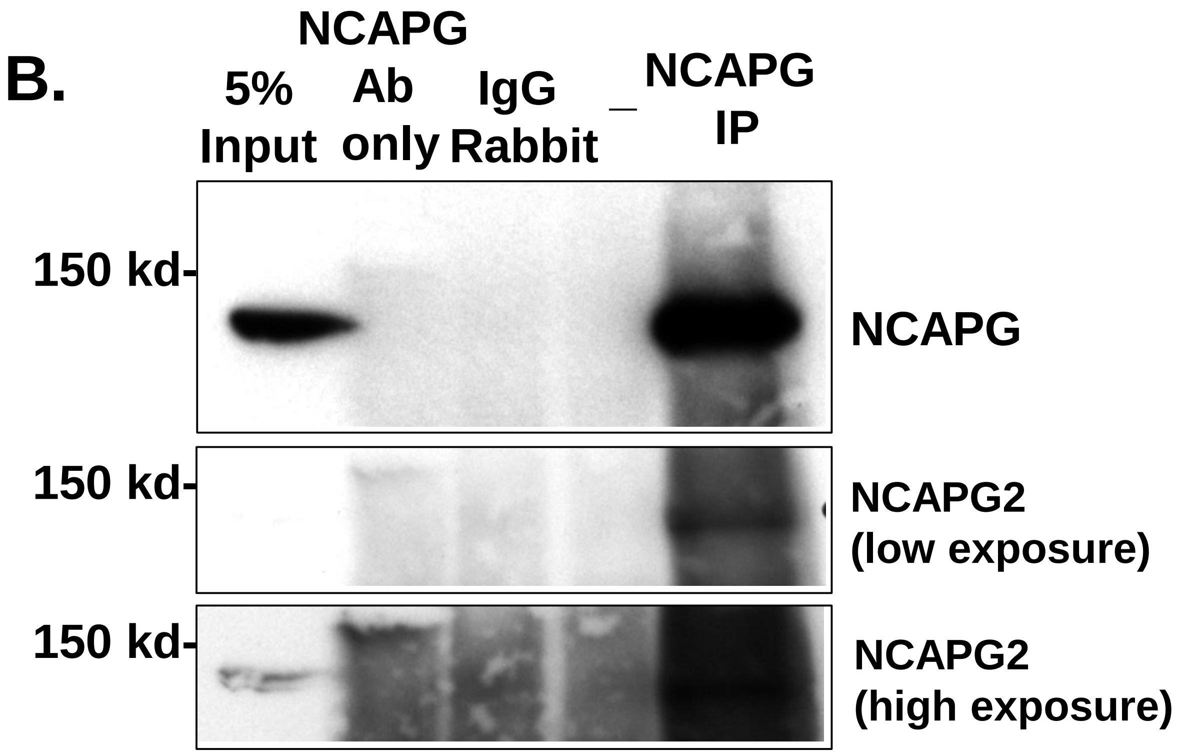

Ward Supp Fig. 8

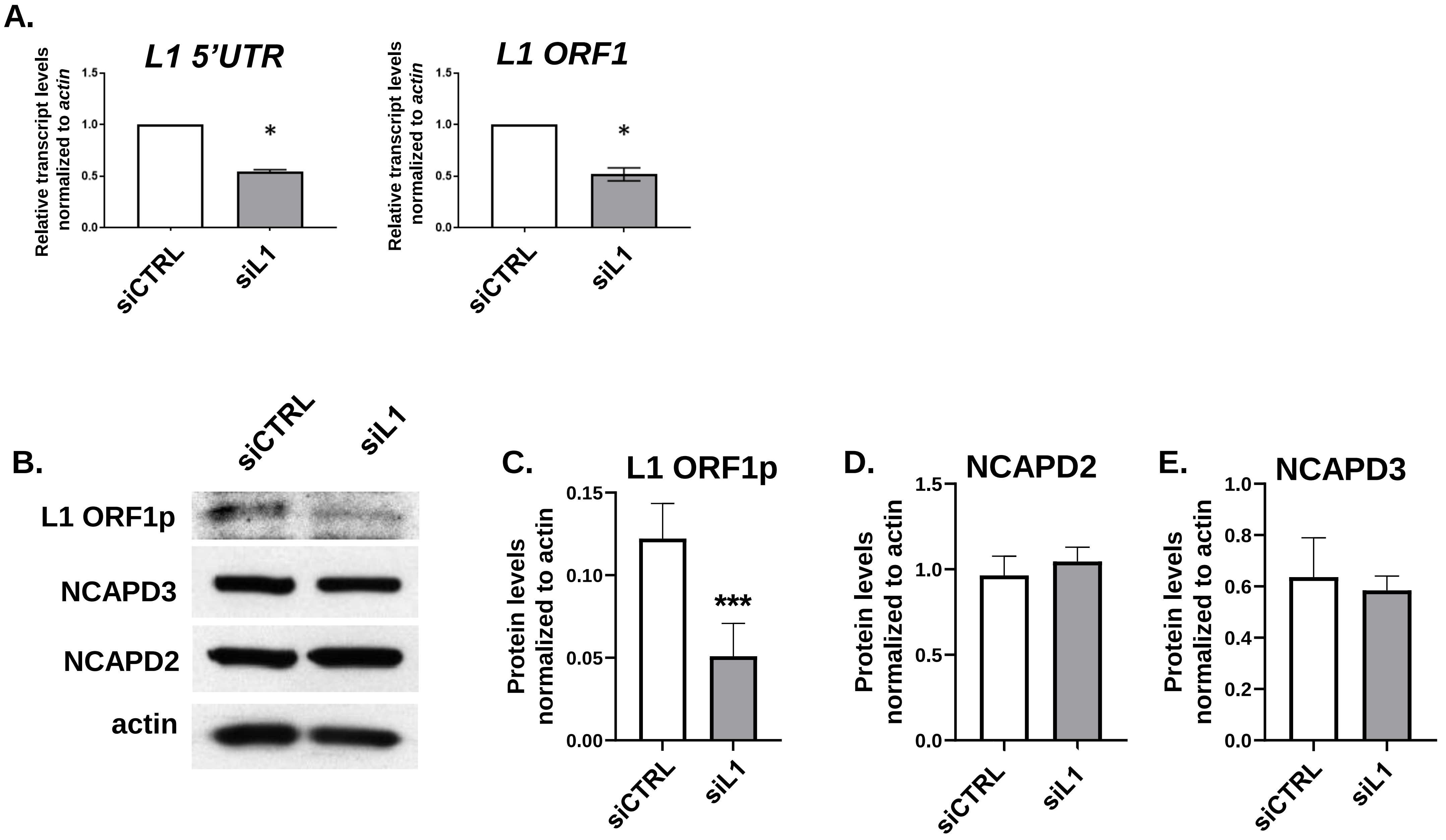

Ward Supp Fig. 9

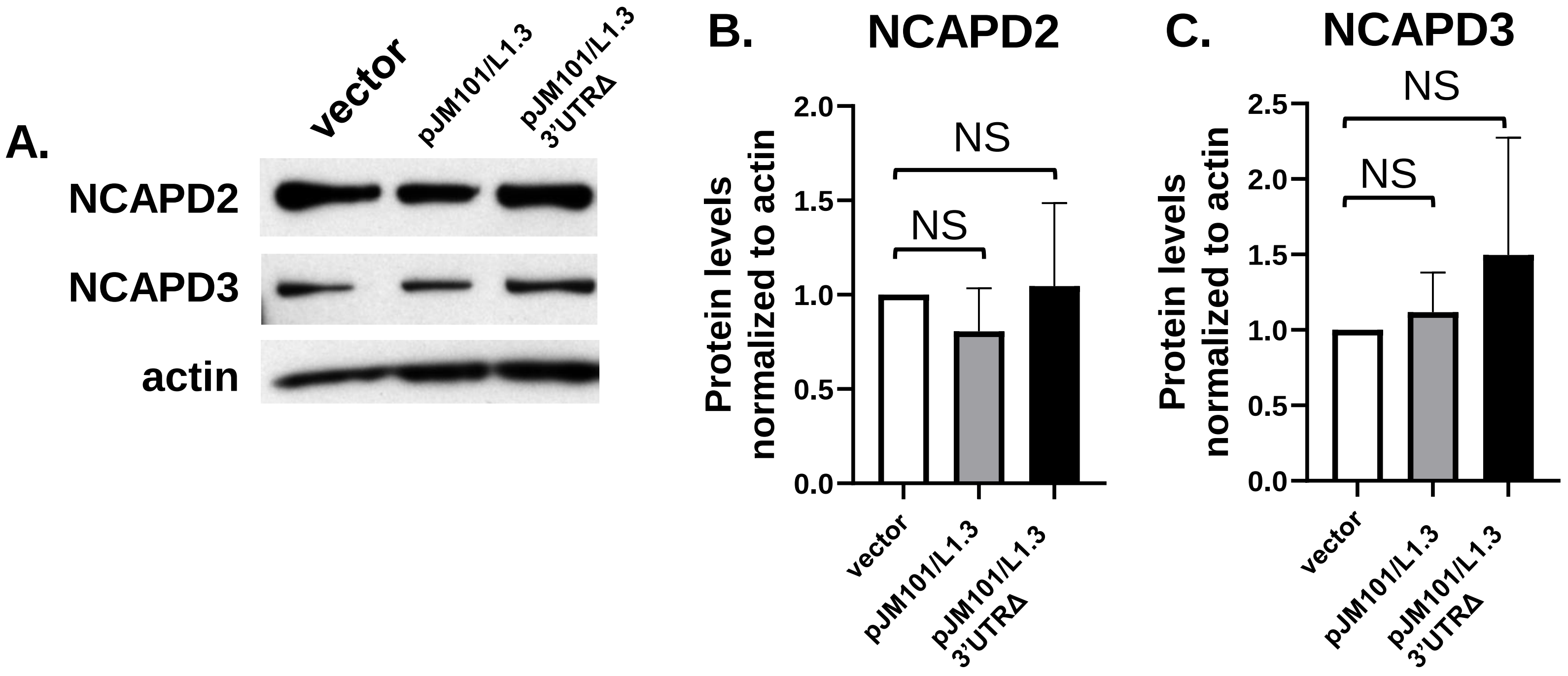

Ward SUPP Fig. 10

Supplemental Figure Legends

Supplemental Figure 1. Diagram of L1 oligonucleotide primer locations and the pJM101L1.3 3'UTR sequence. *L1 5'UTR* and *L1 ORF1* primer sets were used in qRT-PCR assays to detect transcripts from endogenous and/or transfected L1 sequences. An *L1 NEO* primer set was used in RIP-qRT-PCR assays to detect transcripts produced from engineered L1 expression constructs (pJM101/L1.3 and pJM101/L1.3 3'UTRΔ). The sequences of the primers are included in the Methods section. Nucleotides deleted from the 3'UTR to create pJM101L1.3 3'UTRΔ are shown in blue, and nucleotides added following cloning procedures are shown in red.

Supplemental Figure 2. Expression of pJM101L1.3 3'UTRΔ does not affect NCAPD3 or EPRS immunoprecipitation. (A, B) RNA-IPs for (A) NCAPD3 and (B) EPRS were performed in HT-29 cells transfected with either pJM101/L1.3 or pJM101/L1.3 3'UTRΔ. Levels of immunoprecipitated protein were analyzed by immunoblot. RNA-IPs conducted with no antibody (beads only) or with an IgG antibody served as negative controls. Band intensities were quantified from film and results for protein levels in pJM101/L1.3 transfected cells were set to 1. **(C, D)** Quantification of three independent experiments is shown in (C) and (D). P values were determined by performing unpaired T-tests. NS=not significant. Error bars indicate standard deviations from the mean**.**

Supplemental Figure 3. Knockdown of NCAPD2 does not affect the protein levels of condensin II proteins. (A) Immunoblotting for NCAPD2 (top panel) and SMC2 (bottom panel) was performed using whole cell lysates from HT-29 cells expressing Non-target (NT) shRNA or NCAPD2 shRNA. **(B)** Immunoblotting for SMC4 (top panel) and NCAPG2 (bottom panel) was performed using whole cell lysates from HT-29 cells expressing Non-target (NT) shRNA or NCAPD2 shRNA. **(C)** Immunoblotting for NCAPD3 (top panel) and NCAPH2 (bottom panel) was performed using whole cell lysates from HT-29 cells expressing Non-target (NT) shRNA or NCAPD2 shRNA. Band intensities in (A-C) were quantified from film; protein levels were normalized to tubulin. Quantification of three independent experiments is shown to the right and protein levels in cells expressing NT shRNA were set to 1. P values were determined by performing unpaired T-tests. *$p \leq 0.05$, NS= not significant. Error bars indicate standard deviations from the mean**.**

Supplemental Figure 4. Knockdown of NCAPG does not affect the protein levels of Condensin II proteins. (A) Immunoblotting for NCAPG (top panel), NCAPG2 (second panel), SMC2 (third panel), and NCAPD3 (bottom panel) was performed using whole cell lysates from HT-29 cells expressing Non-target (NT) shRNA or NCAPG shRNA. **(B)** Immunoblotting for NCAPH2 was performed using whole cell lysates from HT-29 cells expressing Non-target (NT) shRNA or NCAPG shRNA. **(C)** Immunoblotting for SMC4 was performed using whole cell lysates from HT-29 cells expressing Non-target (NT) shRNA or NCAPG shRNA. Band intensities in (A-C) were quantified from film; protein levels were normalized to tubulin. Quantification of three independent experiments is shown to the right and protein levels in cells expressing NT shRNA were set to 1. P values were determined by performing unpaired T-tests. ****$p \leq 0.0001$, NS= not significant. Error bars indicate standard deviations from the mean**.**

Supplemental Figure 5. Expression of pJM101L1.3 3'UTRΔ does not affect NCAPD2 immunoprecipitation. (A-B) RNA-IPs for NCAPD2 were performed in HT-29 cells transfected with either pJM101/L1.3 or pJM101/L1.3 3'UTRΔ. Levels of immunoprecipitated protein were analyzed by immunoblotting. RNA-IPs with no antibody (beads only) or with an IgG antibody served as negative controls. Band intensities were quantified from film and results for protein levels in pJM101/L1.3 transfected cells were set to 1. Quantification of three independent experiments is shown in (B). P values were determined by performing unpaired T-tests. NS=not significant. Error bars indicate standard deviations from the mean.

Supplemental Figure 6. Condensin I is not required for the association between EPRS/GAIT and NCAPD3/condensin II. IP/immunoblotting experiments for EPRS and NCAPD3 were performed in Non-Target and NCAPD2 shRNA expressing HT-29 cells. Antibody only (no lysate) and IgG antibody IPs served as negative controls. The average amount of immunoprecipitated NCAPD3 protein, normalized to the amount of immunoprecipitated EPRS protein, was quantified in both cell lines. P values were determined by performing unpaired T-tests. NS=not significant. Two independent experiments were performed in the analysis.

Supplemental Figure 7. Expression of NCAPD2 shRNA does not affect NCAPD3, EPRS, or eIF4G immunoprecipitation. (A-C) RNA-IPs for (A) NCAPD3, (B) EPRS and (C) eIF4G were performed in HT-29 cells transfected with pJM101/L1.3. Levels of immunoprecipitated protein were analyzed by immunoblotting. RNA-IPs with no antibody (beads only) or with an IgG antibody served as negative controls. Band intensities were quantified from film and results for protein levels in pJM101/L1.3 transfected cells were set to 1. Quantification of three independent experiments is shown in the charts to the right. P values were determined by performing unpaired T-tests. *$p \leq 0.05$, NS=not significant. Error bars indicate standard deviations from the mean.

Supplemental Figure 8: NCAPG co-precipitates NCAPD3 and NCAPG2. (A-B) NCAPG co-IP/immunoblots were conducted in HT-29 cells to detect an association with (A) NCAPD3 and with (B) NCAPG2. Antibody only (*i.e.*, no lysate) and IgG antibody IPs served as negative controls. Each experiment was performed three times, and representative blots are shown.

Supplemental Figure 9. L1 siRNA effectively decreases endogenous L1 transcripts and protein. (A) Quantitative RT-PCR analysis of L1 RNA levels in HT-29 cells transfected with control (siCTRL) or L1 siRNA (siL1). Shown is the average of three independent experiments. **(B)** HT-29 cells were transfected with control or L1 siRNA and aliquots from whole cell lysates were subjected to immunoblot analysis using antibodies against NCAPD2, NCAPD3, L1 ORF1p, or actin (which served as a loading control). **(C-E)** Band intensities were quantified from film and normalized to actin. Experiments in panel C were performed five times and experiments in panels D and E were performed three times. P values were determined by performing unpaired T-tests. *$p \leq 0.05$. ***$p \leq 0.001$. Error bars indicate standard deviations from the mean.

Supplemental Figure 10: The expression of engineered L1s does not significantly affect the levels of NCAPD2 or NCAPD3 proteins. (A) HT-29 cells were transfected

with either the pCEP4 empty vector, pJM101/L1.3 expression vector, or L1 expression vector harboring a 3'UTR deletion (pJM101L1.3/Δ3'UTR). Aliquots from whole cell lysates were then subjected to immunoblot analysis using antibodies against NCAPD2, NCAPD3, or actin (which served as a loading control). **(B-C)** Quantification of NCAPD2 (B) and NCAPD3 (C) protein levels. Band intensities were quantified from film and normalized to actin. Averages from three independent experiments are shown. P values were determined by performing unpaired T-tests. NS=not significant. Error bars indicate standard deviations from the mean**.**
